# Effects of wogonoside on the inflammatory response and oxidative stress in mice with nonalcoholic fatty liver disease

**DOI:** 10.1080/13880209.2020.1845747

**Published:** 2020-11-30

**Authors:** Guangyu Jiang, Dayin Chen, Wenpeng Li, Chengcheng Liu, Jiguang Liu, Yingxue Guo

**Affiliations:** a College of Pharmacy, Jiamusi University, Jiamusi, China; b Department of Neurosurgery, Shenzhen SAMII Medical Center, Shenzhen, China; c Heilongjiang Agricultural Vocational and Technical College, Jiamusi, China

**Keywords:** Inflammation, NF-κB pathway, Nrf2/HO-1 pathway

## Abstract

**Context:**

Wogonoside has many pharmacological activities, but whether it has a protective effect against non-alcoholic fatty liver disease (NAFLD) has not been reported.

**Objective:**

This study investigates the protective effect of wogonoside against NAFLD in mice and its potential mechanism.

**Materials and methods:**

C57BL/6 mice were randomly divided into control group, NAFLD group and low-, medium- and high-dose wogonoside groups (5, 10 and 20 mg/kg, respectively) (*n*= 12). Mice in the control group were fed with the standard diet, and those in NAFLD group and low-, medium- and high-dose wogonoside groups were fed with a high-fat diet. The different doses of wogonoside were administered by gavage once a day for 12 weeks.

**Results:**

Compared with those in NAFLD group, the liver mass, liver index and the LDL, TG, TC, IL-2, IL-6, TNF-α, MDA and NF-κB p65 levels were decreased, and the SOD and GSH-Px activities, and HDL, IκBα, Nrf2 and HO-1 contents were increased in wogonoside groups. Compared with those in the NAFLD group, wogonoside (5, 10 and 20 mg/kg) reduced AST (132.21 ± 14.62, 115.70 ± 11.32 and 77.94 ± 8.86 vs. 202.35 ± 19.58 U/L) and ALT (104.37 ± 11.92, 97.53 ± 10.12 and 56.74 ± 6.33 vs. 154.66 ± 14.23 U/L) activities in the serum.

**Discussion and conclusions:**

Wogonoside has a protective effect against NAFLD in mice, which may be related to its anti-inflammation and inhibition of oxidative stress, suggesting that wogonoside may be a potential therapeutic agent for the treatment of NAFLD.

## Introduction

Non-alcoholic fatty liver disease (NAFLD) is characterized by the excessive fat deposition in the liver and the steatosis of liver cells (Boutari and Mantzoros 2020). With the change of people's eating habits and the improvement of their living standards, the population of obesity, hypertension, hyperlipidaemia and non-insulin-dependent diabetes is increasing, so the risk of people suffering from NAFLD is also increasing year by year and then NAFLD will further induce the occurrence of cardiovascular and cerebrovascular events (Danford and Lai 2019). NAFLD has posed a serious threat to social development and human health, and is a new major health problem.

**Figure 1.**
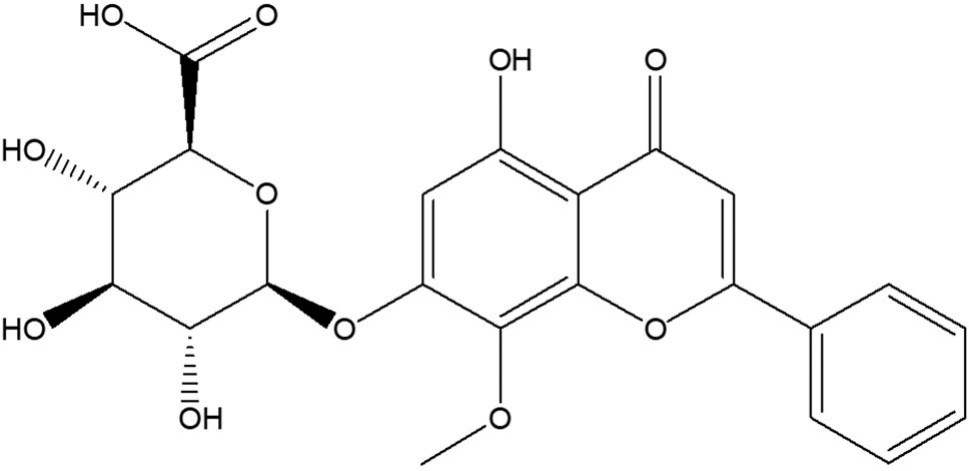
Chemical structure of wogonoside.

Wogonoside, a flavonoid monomer (Figure 1), is one of the active components of traditional Chinese medicine *Scutellaria baicalensis* Georgi (Scutellaria) (Yan et al. 2020). Wogonoside has many pharmacological activities, such as neuroprotection, cardiovascular protection, antidiabetic, antioxidative, antibacterial, anti-inflammatory and antitumor (Ku and Bae 2014; Zhang et al. 2014; Chen et al. 2019). It has been found that wogonoside can protect against the acute liver injury induced by lipopolysaccharide (LPS) and d-galactosamine (d-GalN) by activating Nrf2 and inhibiting the activation of NLRP3 inflammasome (Gao et al. 2016). In addition, wogonoside also has a significant protective effect against CCl_4_-induced liver fibrosis (Wang et al. 2015). So far, the therapeutic effect of wogonoside on NAFLD has not been reported. Given the importance of inflammation and oxidative stress in NAFLD (Pan and An 2018; Farzanegi et al. 2019), the current study explored the effects of wogonoside on the inflammation and oxidative stress in mice induced by a high-fat diet.

## Materials and methods

### Drugs and reagents

Wogonoside (purity > 90.0%) was purchased from DESITE Pharmaceutical Co., Ltd., Chengdu, China; serum aspartate aminotransferase (AST), alanine aminotransferase (ALT), high density lipoprotein (HDL), low density lipoprotein (LDL), triglyceride (TG) and total cholesterol (TC) detection kits were purchased from Yuanye Biological Co. Ltd., Shanghai, China; interleukin-2 (IL-2), IL-6, tumour necrosis factor-α (TNF-α), superoxide dismutase (SOD), glutathione peroxidase (GSH-Px) and malonyldialdehyde (MDA) detection kits were purchased from Jiancheng Bioengineering Institute of Nanjing, Nanjing, China; anti-NF-κB p65, anti-IκBα, anti-Nrf2, anti-HO-1 and anti-β-actin primary antibodies were purchased from Cell Signalling Technology, Beverly, MA. All other reagents were of analytical grade.

### Animals

Male C57BL/6 mice, aged 4 weeks, were purchased from Beijing Weitonglihua Company (clean grade; licence no.: SYXK (Beijing, China) 2017-0025). They were housed in 50% relative humidity and at 23 °C according to the national standards for experimental animals. The animal experiments were carried out in a manner that was consistent with the provisions of China Animal Welfare Act and the Guide of NIH Experimental Animal Management and Use after being approved by the Ethical Committee of Experimental Animals of Jiamusi University.

### Animal grouping, modelling and administration

The male C57BL/6 mice were randomly divided into control group, NAFLD group and low-, medium- and high-dose wogonoside groups (5, 10 and 20 mg/kg, respectively) (*n*= 12). The mice in the control group were fed with the standard diet, and those in the NAFLD group and low-, medium and high-dose wogonoside groups with a high-fat diet (0.5% cholic acid, 2% cholesterol and 15% lard in the standard diet). The mice were administered the corresponding doses of drugs by gavage once a day for 12 weeks.

### Pathological observation of liver tissue

At the end of the last administration, two mice in each group were randomly selected and sacrificed by cervical dislocation. The liver samples were perfused with sterile saline to remove the blood cells and fixed in 10% formaldehyde solution for 24 h, then dehydrated, hyalinized, immersed in wax and paraffin-embed and continuously sliced into 5 μm thick slices by a microtome. The slices were dried for use. The dried slices were stained by haematoxylin–eosin (HE) and mounted for the preparation of sections. The sections were examined under a light microscope for the observation of the pathological changes of liver tissue.

### Measurement of body mass, liver mass and liver index

At the end of the last administration, the body mass of the remaining mice in each group was weighed. The blood samples were collected by removing their eyeballs, and then the mice were sacrificed by cervical dislocation. The liver was separated and weighed accurately, and the liver index was calculated according to the following formula.

Liver index = liver mass/body mass × 100%

### Determination of serum biochemical indicators

The blood was collected in the same way as described above and 1.5 mL of the blood sample was placed into a centrifuge tube and centrifuged at 3000 rpm for 10 min to obtain the serum. AST and ALT activities and HDL, LDL, TG and TC contents in the serum of mice were detected by an automatic biochemical analyser.

### Measurement of inflammatory factors and oxidative stress-related indicators in the liver tissue

The liver tissue (100 μg) of mice taken from the same part was placed into a cup containing 0.9 mL cold sterile saline and homogenized on ice, and then the liver tissue-saline solution was centrifuged (3000 rpm for 5 min at 4 °C) to prepare a homogenate containing 10% of the liver tissue. The contents of IL-2, IL-6 and TNF-α in the liver tissue were determined by enzyme-linked immunosorbent assay (ELISA), and the SOD and GSH-Px activities and the MDA contents in the liver tissue were determined by spectrophotometry.

### Western blot analysis

The total protein and nucleoprotein of liver tissue were extracted, respectively. The proteins were quantified by BCA method and denatured in a boiling water bath, and then the SDS gel electrophoresis on the proteins was performed. The proteins were transferred onto PVDF membranes by semi-dry method, then the first antibodies (1:1000) were added onto the membranes after the blocking with the skimmed milk powder for 2 h and the membranes were incubated at 4 °C overnight. The membranes were washed three times with TBST and then incubated with the corresponding second antibody (1:2000) labelled by HRP for 2 h. ECL was used for the colour development and the films were exposed in a darkroom. The images were scanned and the absorbance value of each band was analysed with Image J software (Bethesda, MD), in which β-actin was used as the internal reference for the standard control of the relative expression calculation of the target proteins.

### Statistical methods

SPSS 20.0 software (SPSS Inc., Chicago, IL) was used to analyse the data. The measurement data were expressed as ‘mean ± SD’ and the differences between the mean values were analysed for significance using a one-way analysis of variance (ANOVA). *p* < 0.05 or *p* < 0.01 indicated that the difference was a highly significant, respectively.

## Results

### Effects of wogonoside on the body mass, liver mass, liver index and serum AST and ALT activities in NAFLD mice

**Figure 2.**
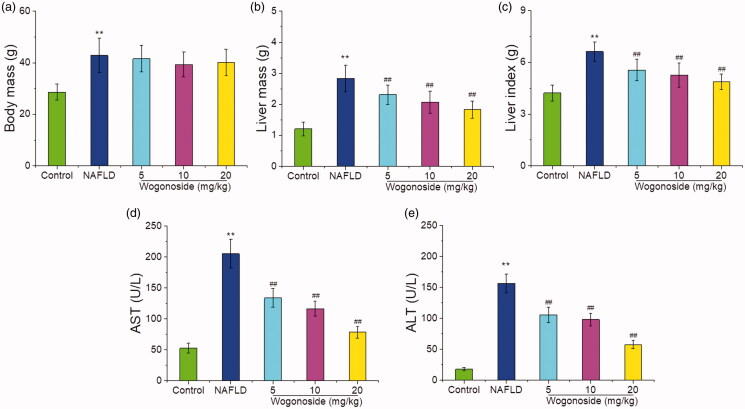
Effects of wogonoside on the body mass (a), liver mass (b), liver index (c) and the serum AST (d) and ALT (e) activities in NAFLD mice. Values are expressed as mean ± SD, *n*= 10. Compared with the control group: **p< 0.01; Compared with NAFLD group: ^##^*p*< 0.01.

In order to verify the protective effect of wogonoside against the liver injury in NAFLD mice, the effects of wogonoside on the body mass, liver mass, liver index and AST and ALT activities in the serum of NAFLD mice were detected. The results showed that compared with those in the control group, the body mass, liver mass and liver index of mice in NAFLD group were significantly higher (*p* < 0.01), suggesting that the NAFLD model was successfully established; compared with that in NAFLD group, the body mass of mice in wogonoside groups was not significantly different (*p*> 0.05), but the liver mass and liver index were significantly lower, respectively (*p* < 0.01) (Figure 2(a–c)). Compared with those in the control group, the serum AST (202.35 ± 19.58 vs. 53.48 ± 7.91 U/L) and ALT (154.66 ± 14.23 vs. 17.84 ± 1.79 U/L) activities of mice in NAFLD group were significantly increased (*p* < 0.01), and compared with those in NAFLD group, the serum AST (132.21 ± 14.62, 115.70 ± 11.32 and 77.94 ± 8.86 vs. 202.35 ± 19.58 U/L) and ALT (104.37 ± 11.92, 97.53 ± 10.12 and 56.74 ± 6.33 vs. 154.66 ± 14.23 U/L) activities of mice in wogonoside groups were significantly decreased (*p* < 0.01) (Figure 2(d,e)), suggesting that wogonoside could have a protective effect against the liver injury of NAFLD mice.

### Effects of wogonoside on the pathological morphology of liver in NAFLD mice

**Figure 3.**
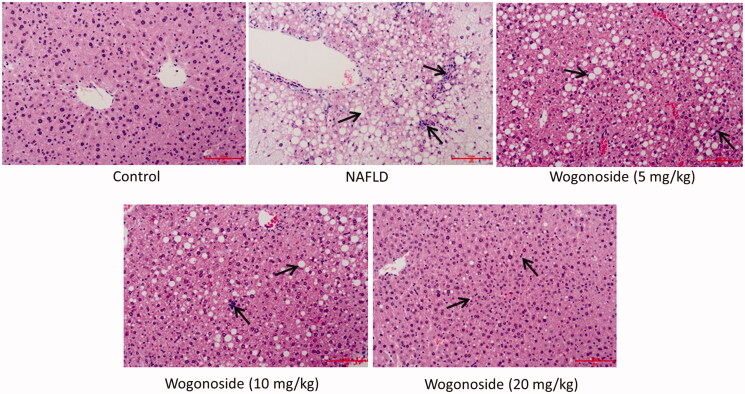
Effects of wogonoside on the pathological morphology of liver tissue in NAFLD mice (HE, ×200).

HE staining was used to detect the effect of wogonoside on the pathological morphology of liver tissue in NAFLD mice. It was found that in the control group, the structure of liver lobules was clear, the structure of liver tissue was normal, the surrounding cell cords were radially arranged and there was no cell with fatty degeneration; in NAFLD group, the boundary of liver lobules was not clear, the structure of liver tissue showed some pathological changes, such as a large number of globular lipid droplets and inflammatory cells infiltrated; compared with NAFLD group, the pathological morphology of liver tissue was improved to some extent in the low-, medium- and high-dose wogonoside groups, such as the reduced globular lipid droplets and infiltration of inflammatory cells, especially in the high-dose wogonoside group, as shown in Figure 3.

### Effects of wogonoside on the serum HDL, LDL, TG and TC contents of in NAFLD mice

**Figure 4.**
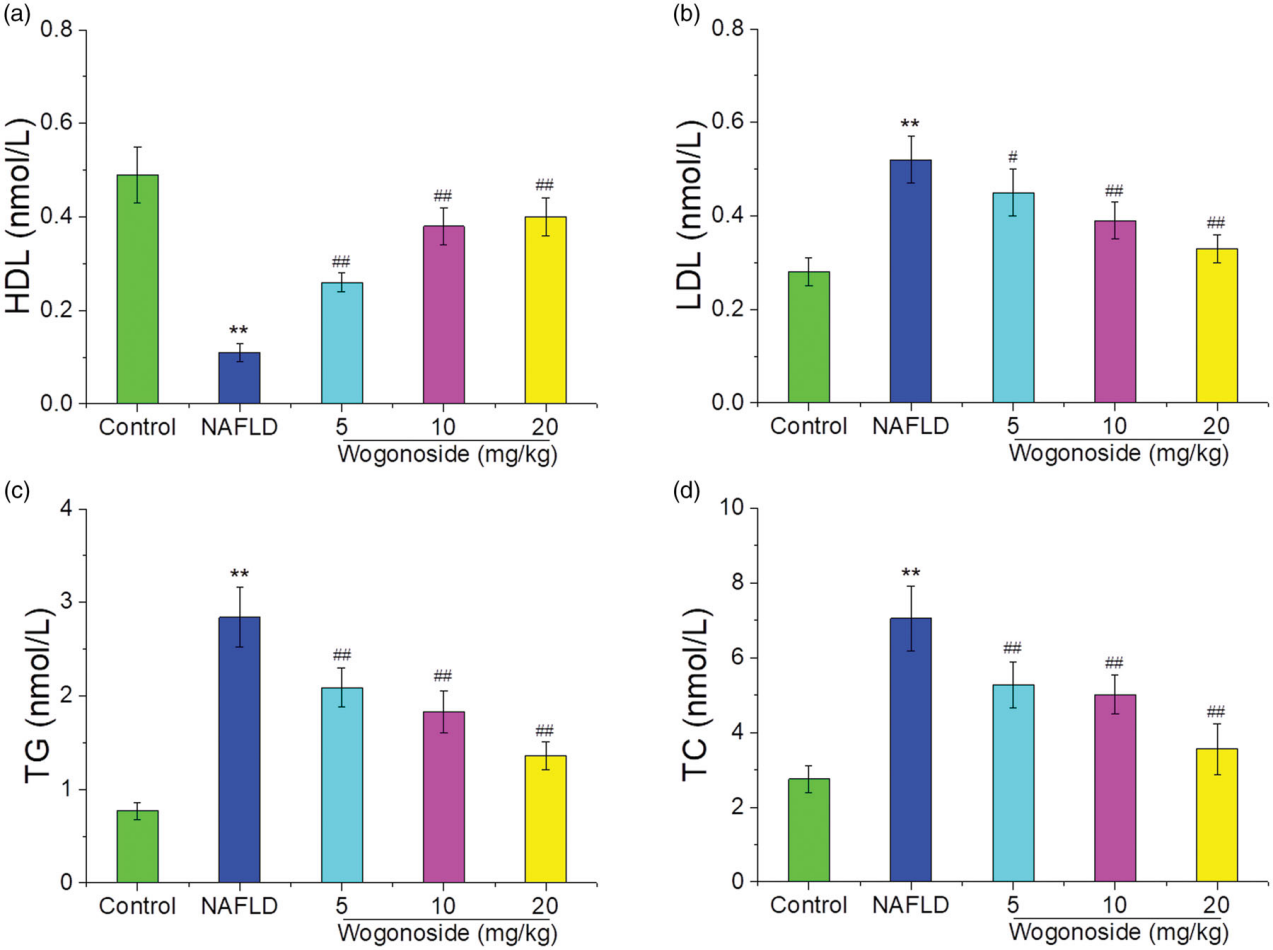
Effects of wogonoside on the serum HDL (a), LDL (b), TG (c) and TC (d) contents in NAFLD mice. Values are expressed as mean ± SD, *n*= 10. Compared with the control group: **p< 0.01; Compared with NAFLD group: ^#^*p*< 0.05, ^##^*p*< 0.01.

As shown in Figure 4, compared with those in the control group, the serum HDL contents were significantly decreased, while the serum LDL, TG and TC contents were significantly increased in NAFLD group (*p* < 0.01); compared with those in NAFLD group, the serum HDL contents were significantly increased, while the serum LDL, TG and TC contents were significantly decreased (*p* < 0.05 or *p* < 0.01), suggesting that wogonoside could play a protective role against the liver injury by regulating the lipid metabolism disorder in NAFLD mice.

### Effects of wogonoside on the inflammatory response of liver tissue in NAFLD mice

**Figure 5.**
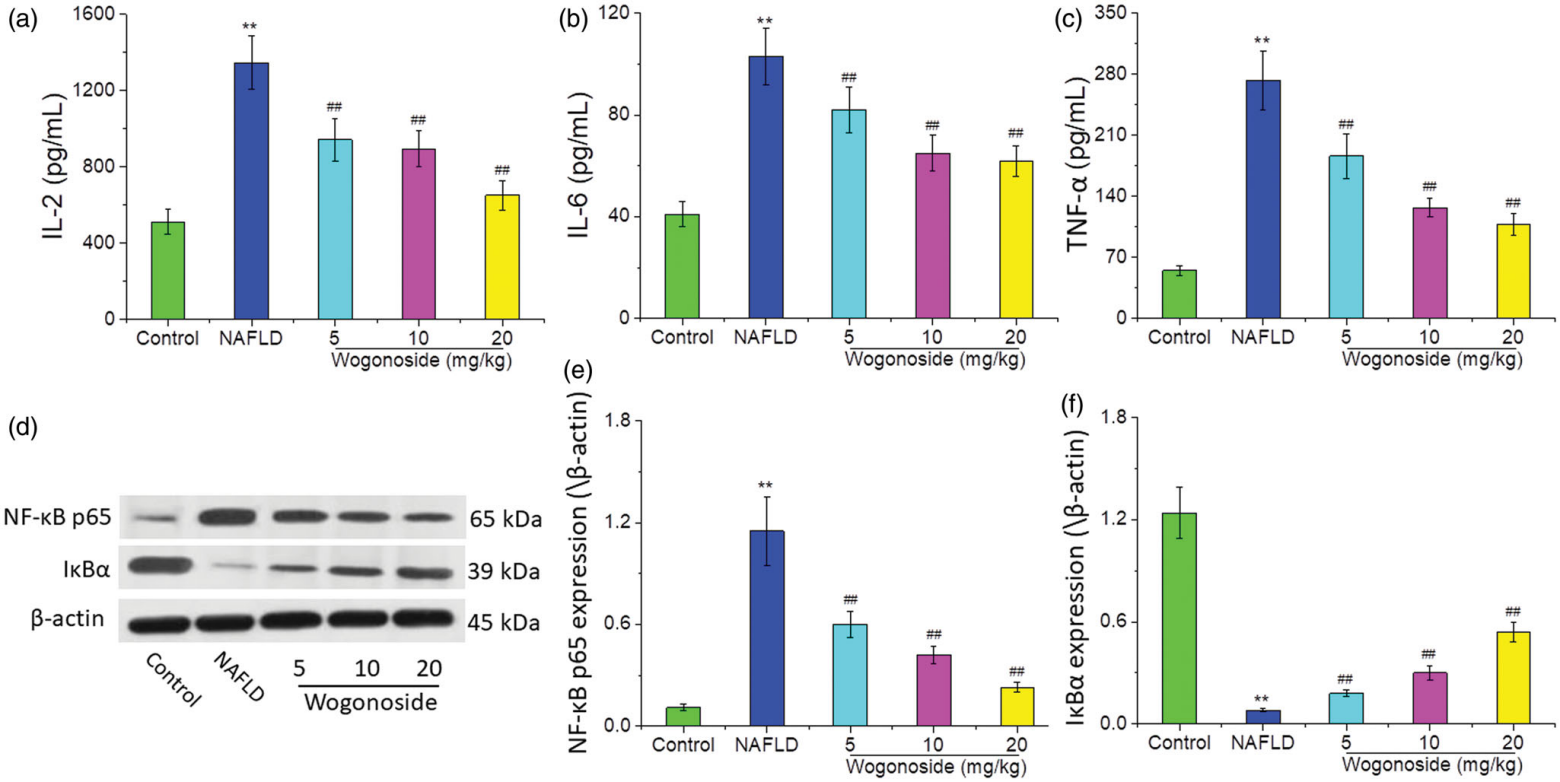
Effects of wogonoside on the inflammatory response of liver tissue in NAFLD mice. (a) IL-2 levels; (b) IL-6 levels; (c) TNF-α levels; (d) Western blot analysis; (e) protein relative expression of NF-κB p65; (f) protein relative expression of IκBα. Values are expressed as mean ± SD, *n*= 10. Compared with the control group: **p< 0.01; Compared with NAFLD group: ^##^*p*< 0.01.

Compared with those in the control group, the contents of IL-2 (1345.28 ± 123.30 vs. 510.73 ± 56.43 pg/mL), IL-6 (101.81 ± 10.54 vs. 40.76 ± 5.73 pg/mL) and TNF-α (270.30 ± 31.48 vs. 54.50 ± 5.14 pg/mL) in the liver tissue of mice in NAFLD group increased significantly (*p* < 0.01), and compared with those in NAFLD group, the contents of IL-2 (940.86 ± 107.47, 886.71 ± 90.69 and 649.92 ± 68.11 vs. 1345.28 ± 123.30 pg/mL), IL-6 (81.37 ± 8.26, 63.34 ± 7.71 and 60.83 ± 5.08 vs. 101.81 ± 10.54 pg/mL) and TNF-α (184.04 ± 19.20, 125.79 ± 10.65 and 106.74 ± 11.48 vs. 270.30 ± 31.48 pg/mL) in the liver tissue of mice in the wogonoside-treated groups decreased significantly (*p* < 0.01), as shown in Figure 5(a–c). Western blot analysis showed that compared with that in the control group, the expression of NF-κB p65 in the liver tissue of mice in NAFLD group increased significantly, while that of IκBα decreased significantly (*p* < 0.01), and compared with that in NAFLD group, the expression of NF-κB p65 in the liver tissue of mice decreased significantly, while that of IκBα increased significantly in the wogonoside-treated groups (*p* < 0.01), as shown in Figure 5(d–f). The results suggest that wogonoside can protect NAFLD mice from the liver injury by regulating the NF-κB pathway to inhibit the inflammation.

### Effect of wogonoside on the oxidative stress level of liver tissue in NAFLD mice

**Figure 6.**
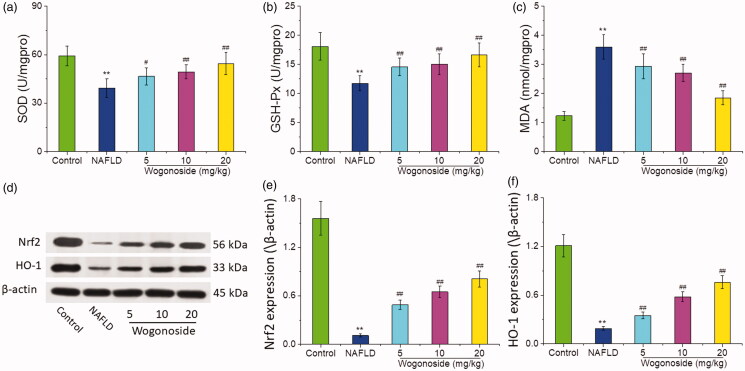
Effect of wogonoside on the oxidative stress level of liver tissue in NAFLD mice. (a) SOD activities; (b) GSH-Px activities; (c) MDA levels; (d) Western blot analysis; (e) protein relative expression of Nrf2; (f) protein relative expression of HO-1. Values are expressed as mean ± SD, *n*= 10. Compared with the control group: **p< 0.01; compared with NAFLD group: ^#^p < 0.05, ^##^p < 0.01.

Compared with that in the control group, the activities of SOD (37.34 ± 4.76 vs. 59.63 ± 6.21 U/mg protein) and GSH-Px (12.68 ± 1.08 vs. 16.21 ± 2.17 U/mg protein) decreased significantly, while the content of MDA (3.61 ± 0.40 vs. 1.24 ± 0.13 nmol/mg protein) increased significantly in the liver tissue of mice in NAFLD group (*p* < 0.01); compared with that in NAFLD group, the activities of SOD (45.57 ± 5.09, 48.21 ± 4.30 and 53.35 ± 5.97 vs. 37.34 ± 4.76 U/mg protein) and GSH-Px (14.46 ± 1.53, 15.09 ± 1.76 and 16.31 ± 2.04 vs. 12.68 ± 1.08 U/mg protein) increased significantly, while the content of MDA (2.95 ± 0.33, 2.70 ± 0.25 and 1.86 ± 0.21 vs. 3.61 ± 0.40 nmol/mg protein) decreased significantly in the low-, medium- and high-dose wogonoside groups (*p* < 0.05 or *p* < 0.01), as shown in Figure 6(a–c). Western blot analysis showed that the expression of Nrf2 and HO-1 proteins in the liver tissue of mice in NAFLD group was significantly lower than that in the control group (*p* < 0.01), and the expression of Nrf2 and HO-1 proteins in the liver tissue of mice in the low-, medium- and high-dose wogonoside groups was significantly higher than that in NAFLD group (*p* < 0.01), as shown in Figure 6(d–f). These results suggest that wogonoside can protect NAFLD mice from the liver injury by regulating the Nrf2/HO-1 pathway to inhibit the oxidative stress.

## Discussion

Fat metabolism mainly occurs in the liver and a large amount of high-fat diet for a long time will increase the fat intake of the liver, and when the rate of fat intake in the liver is greater than that of its excretion, the fatty degeneration in the liver will be induced (Kashyap et al. 2019). The animal model induced by high-fat diet can better simulate the occurrence and development of human non-alcoholic fatty liver, with the characteristics of insulin resistance and obesity, and is widely used in the related studies (Braud et al. 2017). In this study, mice were fed on a high-fat diet for 12 weeks for preparing a model of NAFLD mice. The results showed that the body mass, liver mass, liver index and serum AST and ALT activities of mice in the NAFLD model group were significantly increased, and there were a large number of lipid droplets and inflammatory infiltrating cells in the liver tissue of NAFLD mice, suggesting that the NAFLD mouse model was successfully established, while the liver mass, liver index and the activities of AST and ALT in the serum of mice in the wogonoside-treated groups were significantly lower than those in NAFLD group, and the pathological changes of the liver were also improved in wogonoside-treated groups, indicating that wogonoside could protect the liver of NAFLD mice. The serum HDL, LDL, TG and TC contents are the most commonly used indexes for NAFLD (Liang et al. 2018). The results of this study show that wogonoside could reduce the content of serum HDL, LDL and TG in NAFLD mice, and increase the content of HDL, further confirming the protective effect of wogonoside against the liver injury in NAFLD mice.

Inflammation plays an important role in the occurrence and development of NAFLD, and IL-2, IL-6 and TNF-α are closely related to the occurrence of inflammation (Oliveira et al. 2019). Inflammatory factors, such as IL-2, IL-6 and TNF-α, can promote the steatosis of hepatocytes, and eventually lead to NAFLD (Ezquerro et al. 2019). *In vitro* and *in vivo* studies show that wogonoside could reduce the expression of TNF-α, IL-6 and IL-1 and improve the symptoms of osteoarthritis in mice (Tang et al. 2017). It was also found in this study that the content of IL-2, IL-6 and TNF-α in the liver tissue of NAFLD mice increased significantly, while that of IL-2, IL-6 and TNF-α in the wogonoside groups decreased significantly, suggesting that wogonoside could effectively inhibit the inflammatory response of liver tissue of NAFLD mice. NF-κB pathway plays a key role in inflammatory response, mediating the transcription and expression of many inflammatory factors (Deng et al. 2018). Normally, NF-κB exists in the cytoplasm and is activated by some signals, such as oxidative stress, then the inhibitory subunit IкB will be dissociated and the functional subunits such as p65 subunit will be translocated into the nucleus to play the role of transcriptional regulation (Zeng et al. 2015; Sindhu et al. 2019). Previous studies found that wogonoside could promote the glucose uptake of HepG2 cells and increase the glycogen synthesis to improve the liver ischaemia and reperfusion (IR) injury, which may be related to NF-κB inflammatory pathway (Zhu et al. 2019). The results of this study also showed that compared with that in NAFLD group, the expression of NF-κB p65 decreased and that of IκBα increased in the wogonoside-treated groups, further indicating that wogonoside can protect NAFLD mice from the liver injury by regulating the NF-κB pathway and inhibiting the inflammation.

Oxidative stress, one factor leading to the pathogenesis of NAFLD, refers to the accelerated production of ROS in the body, which is beyond the body’s ability to scavenge it, leading to the injury of tissues (Yang and Jo 2018). Liver is not only the main site of ROS production, but also the main target organ of ROS attack (Zhang et al. 2020). SOD, an important ROS scavenger, can keep ROS in balance in the body and in NAFLD model, the activity of SOD is reduced and the accumulation of ROS is increased, and finally the injury hepatocytes will be aggravated (Kobyliak et al. 2016). GSH-Px exists in all tissues of human body, among which the activity of GSH-Px is the highest in the liver, and GSH-Px is an important part of endogenous antioxidant system, with the function of clearing ROS and preventing the oxidative stress injury of liver (Niu et al. 2018). MDA, a highly toxic lipid peroxidation product, can spread to the inside and outside of cells, and then aggravate the damage range of an oxidative stress (El-Din et al. 2014). It has been reported that the content of MDA in NAFLD patients increases significantly and is positively correlated with the fatty degeneration, fibrosis, inflammation and necrosis of liver tissue (Świderska et al. 2019). Our results showed that the activities of SOD and GSH-Px decreased significantly, while the content of MDA increased significantly in the liver tissue of mice in NAFLD group, indicating an oxidative damage in the liver tissue; the activities of SOD and GSH-Px increased, while the content of MDA decreased in each wogonoside-treated group significantly, suggesting that wogonoside can effectively inhibit the oxidative stress level of liver tissue in NAFLD mice.

Nrf2 is an important transcription factor regulating antioxidant status, and under normal conditions, Nrf2 and Keap1 form a complex in the cytoplasm to inhibit the activity of Nrf2 (Wu et al. 2019). In the case of oxidative stress, Nrf2 dissociates from Keap1, and Nrf2 translocates into the nucleus to bind to antioxidant response element (ARE) to activate the expression of HO-1, an antioxidant gene, thus enhancing the antioxidant capacity of cells (Fan et al. 2017). The increase of Nrf2 and HO-1 protein expression can enhance the antioxidant capacity of the body and protect the body from the damage of reactive oxygen species (Xu et al. 2018). A number of studies have confirmed that in NAFLD model, the regulation of Nrf2 pathway activation is an important mechanism to play the role of liver protection (Feng et al. 2017; Yan et al. 2018).

The results of this study showed that the expression of Nrf2 and HO-1 in the liver tissue of mice treated with the different doses of wogonoside was significantly increased compared with that in NAFLD group, further confirming that wogonoside can protect NAFLD mice from the liver injury by regulating the Nrf2/HO-1 pathway and inhibiting the oxidative stress.

## Conclusions

Wogonoside has a protective effect against liver injury in NAFLD mice induced by high-fat diet. Regulation of the NF-κB pathway and the inhibition of inflammatory response are mechanisms through which wogonoside can protect NAFLD mice from liver injury. In addition, regulation of the Nrf2/HO-1 pathway and the inhibition of oxidative stress may be another mechanism through which wogonoside can play the protective effect against liver injury in NAFLD mice.

## References

[CIT0001] Boutari C, Mantzoros CS. 2020. Adiponectin and leptin in the diagnosis and therapy of NAFLD. Metabolism. 103:154028. 31785257 10.1016/j.metabol.2019.154028

[CIT0002] Braud L, Battault S, Meyer G, Nascimento A, Gaillard S, Sousa G, Rahmani R, Riva C, Armand M, Maixent JM, et al. 2017. Antioxidant properties of tea blunt ROS-dependent lipogenesis: beneficial effect on hepatic steatosis in a high fat-high sucrose diet NAFLD obese rat model. J Nutr Biochem. 40:95–104. 27866076 10.1016/j.jnutbio.2016.10.012

[CIT0003] Chen S, Wu Z, Ke Y, Shu P, Chen C, Lin R, Shi Q. 2019. Wogonoside inhibits tumor growth and metastasis in endometrial cancer via ER stress-Hippo signaling axis. Acta Biochim Biophys Sin (Shanghai). 51(11):1096–1105. 31696210 10.1093/abbs/gmz109

[CIT0004] Danford CJ, Lai M. 2019. NAFLD: a multisystem disease that requires a multidisciplinary approach. Frontline Gastroenterol. 10(4):328–329. 31682642 10.1136/flgastro-2019-101235 PMC6788273

[CIT0005] Deng Y, Tang K, Chen R, Liu Y, Nie H, Wang H, Zhang Y, Yang Q. 2018. Effects of Shugan-jianpi recipe on the expression of the p38 MAPK/NF-κB signaling pathway in the hepatocytes of NAFLD rats. Medicines (Basel). 5(3):106. 10.3390/medicines5030106 PMC6163402 30235843

[CIT0006] El-Din SH, Sabra AN, Hammam OA, Ebeid FA, El-Lakkany NM. 2014. Pharmacological and antioxidant actions of garlic and or onion in non-alcoholic fatty liver disease (NAFLD) in rats. J Egypt Soc Parasitol. 44(2):295–308. 25597144 10.12816/0006468

[CIT0007] Ezquerro S, Mocha F, Frühbeck G, Guzmán-Ruiz R, Valentí V, Mugueta C, Becerril S, Catalán V, Gómez-Ambrosi J, Silva C, et al. 2019. Ghrelin reduces TNF-α-induced human hepatocyte apoptosis, autophagy, and pyroptosis: role in obesity-associated NAFLD. J Clin Endocrinol Metab. 104(1):21–37. 30137403 10.1210/jc.2018-01171

[CIT0008] Fan H, Ma X, Lin P, Kang Q, Zhao Z, Wang L, Sun D, Cheng J, Li Y. 2017. Scutellarin prevents nonalcoholic fatty liver disease (NAFLD) and hyperlipidemia via PI3K/AKT-dependent activation of nuclear factor (erythroid-derived 2)-like 2 (Nrf2) in rats. Med Sci Monit. 23:5599–5612. 29172017 10.12659/MSM.907530 PMC5712520

[CIT0009] Farzanegi P, Dana A, Ebrahimpoor Z, Asadi M, Azarbayjani MA. 2019. Mechanisms of beneficial effects of exercise training on non-alcoholic fatty liver disease (NAFLD): roles of oxidative stress and inflammation. Eur J Sport Sci. 19(7):994–1003. 30732555 10.1080/17461391.2019.1571114

[CIT0010] Feng X, Yu W, Li X, Zhou F, Zhang W, Shen Q, Li J, Zhang C, Shen P. 2017. Apigenin, a modulator of PPARγ, attenuates HFD-induced NAFLD by regulating hepatocyte lipid metabolism and oxidative stress via Nrf2 activation. Biochem Pharmacol. 136:136–149. 28414138 10.1016/j.bcp.2017.04.014

[CIT0011] Gao YZ, Zhao LF, Ma J, Xue WH, Zhao H. 2016. Protective mechanisms of wogonoside against lipopolysaccharide/d-galactosamine-induced acute liver injury in mice. Eur J Pharmacol. 780:8–15. 26921756 10.1016/j.ejphar.2016.02.040

[CIT0012] Kashyap ML, Ganji S, Nakra NK, Kamanna VS. 2019. Niacin for treatment of nonalcoholic fatty liver disease (NAFLD): novel use for an old drug? J Clin Lipidol. 13(6):873–879. 31706905 10.1016/j.jacl.2019.10.006

[CIT0013] Kobyliak N, Abenavoli L, Falalyeyeva T, Virchenko O, Natalia B, Beregova T, Bodnar P, Spivak M. 2016. Prevention of NAFLD development in rats with obesity via the improvement of pro/antioxidant state by cerium dioxide nanoparticles. Clujul Med. 89(2):229–235. 27152074 10.15386/cjmed-632 PMC4849381

[CIT0014] Ku SK, Bae JS. 2014. Antithrombotic activities of wogonin and wogonoside via inhibiting platelet aggregation. Fitoterapia. 98:27–35. 25020199 10.1016/j.fitote.2014.07.006

[CIT0015] Liang S, Zhang Y, Deng Y, He Y, Liang Y, Liang Z, Chen Y, Tang K, Chen R, Yang Q. 2018. The potential effect of Chinese herbal formula Hongqijiangzhi fang in improving NAFLD: focusing on NLRP3 inflammasome and gut microbiota. Evid Based Complement Alternat Med. 2018:5378961. 29675053 10.1155/2018/5378961 PMC5841032

[CIT0016] Niu T, Xuan R, Jiang L, Wu W, Zhen Z, Song Y, Hong L, Zheng K, Zhang J, Xu Q, et al. 2018. Astaxanthin induces the Nrf2/HO-1 antioxidant pathway in human umbilical vein endothelial cells by generating trace amounts of ROS. J Agric Food Chem. 66(6):1551–1559. 29381356 10.1021/acs.jafc.7b05493

[CIT0017] Oliveira S, Houseright RA, Graves AL, Golenberg N, Korte BG, Miskolci V, Huttenlocher A. 2019. Metformin modulates innate immune-mediated inflammation and early progression of NAFLD-associated hepatocellular carcinoma in zebrafish. J Hepatol. 70(4):710–721. 30572006 10.1016/j.jhep.2018.11.034 PMC6436385

[CIT0018] Pan ZG, An XS. 2018. SARM1 deletion restrains NAFLD induced by high fat diet (HFD) through reducing inflammation, oxidative stress and lipid accumulation. Biochem Biophys Res Commun. 498(3):416–423. 29454967 10.1016/j.bbrc.2018.02.115

[CIT0019] Sindhu S, Kochumon S, Shenouda S, Wilson A, Al-Mulla F, Ahmad R. 2019. The cooperative induction of CCl_4_ in human monocytic cells by TNF-α and palmitate requires MyD88 and involves MAPK/NF-κB signaling pathways. Int J Mol Sci. 20:E4658. 31546972 10.3390/ijms20184658 PMC6770648

[CIT0020] Świderska M, Maciejczyk M, Zalewska A, Pogorzelska J, Flisiak R, Chabowski A. 2019. Oxidative stress biomarkers in the serum and plasma of patients with non-alcoholic fatty liver disease (NAFLD). Can plasma AGE be a marker of NAFLD? Oxidative stress biomarkers in NAFLD patients. Free Radic Res. 53(8):841–850. 31234658 10.1080/10715762.2019.1635691

[CIT0021] Tang Q, Zheng G, Feng Z, Tong M, Xu J, Hu Z, Shang P, Chen Y, Wang C, Lou Y, et al. 2017. Wogonoside inhibits IL-1β induced catabolism and hypertrophy in mouse chondrocyte and ameliorates murine osteoarthritis. Oncotarget. 8(37):61440–61456. 28977876 10.18632/oncotarget.18374 PMC5617436

[CIT0022] Wang Q, Wen R, Lin Q, Wang N, Lu P, Zhu X. 2015. Wogonoside shows antifibrotic effects in an experimental regression model of hepatic fibrosis. Dig Dis Sci. 60(11):3329–3339. 26130019 10.1007/s10620-015-3751-4

[CIT0023] Wu W, Peng G, Yang F, Zhang Y, Mu Z, Han X. 2019. Sulforaphane has a therapeutic effect in an atopic dermatitis murine model and activates the Nrf2/HO-1 axis. Mol Med Rep. 20:1761–1771. 31257541 10.3892/mmr.2019.10405 PMC6625393

[CIT0024] Xu J, Li C, Li Z, Yang C, Lei L, Ren W, Su Y, Chen C. 2018. Protective effects of oxymatrine against lipopolysaccharide/d-galactosamine-induced acute liver failure through oxidative damage, via activation of Nrf2/HO-1 and modulation of inflammatory TLR4-signaling pathways. Mol Med Rep. 17(1):1907–1912. 29138821 10.3892/mmr.2017.8060

[CIT0025] Yan C, Zhang Y, Zhang X, Aa J, Wang G, Xie Y. 2018. Curcumin regulates endogenous and exogenous metabolism via Nrf2-FXR-LXR pathway in NAFLD mice. Biomed Pharmacother. 105:274–281. 29860219 10.1016/j.biopha.2018.05.135

[CIT0026] Yan Y, Yao L, Sun H, Pang S, Kong X, Zhao S, Xu S. 2020. Effects of wogonoside on invasion and migration of lung cancer A549 cells and angiogenesis in xenograft tumors of nude mice. J Thorac Dis. 12(4):1552–1560. 32395292 10.21037/jtd-20-1555 PMC7212121

[CIT0027] Yang DK, Jo DG. 2018. Mulberry fruit extract ameliorates nonalcoholic fatty liver disease (NAFLD) through inhibition of mitochondrial oxidative stress in rats. Evid Based Complement Alternat Med. 2018:8165716. 30643537 10.1155/2018/8165716 PMC6311263

[CIT0028] Zeng C, Zhong P, Zhao Y, Kanchana K, Zhang Y, Khan ZA, Chakrabarti S, Wu L, Wang J, Liang G. 2015. Curcumin protects hearts from FFA-induced injury by activating Nrf2 and inactivating NF-κB both *in vitro* and *in vivo*. J Mol Cell Cardiol. 79:1–12. 25444713 10.1016/j.yjmcc.2014.10.002

[CIT0029] Zhang L, Ren Y, Yang C, Guo Y, Zhang X, Hou G, Guo X, Sun N, Liu Y. 2014. Wogonoside ameliorates lipopolysaccharide-induced acute lung injury in mice. Inflammation. 37(6):2006–2012. 24854163 10.1007/s10753-014-9932-z

[CIT0030] Zhang P, Yin Y, Wang T, Li W, Li C, Zeng X, Yang W, Zhang R, Tang Y, Shi L, et al. 2020. Maresin 1 mitigates concanavalin A-induced acute liver injury in mice by inhibiting ROS-mediated activation of NF-κB signaling. Free Radic Biol Med. 147:23–36. 31785331 10.1016/j.freeradbiomed.2019.11.033

[CIT0031] Zhu SL, Wu QH, Tu J. 2019. Study on regulation of NLRP3/SOCS3-TLR4-NF-κB inflammatory pathway by wogonoside to improve hepatic insulin resistance. Zhongguo Zhong Yao Za Zhi. 44:4504–4510. 31872639 10.19540/j.cnki.cjcmm.20190312.002

